# Three-dimensional single particle tracking using 4π self-interference of temporally phase-shifted fluorescence

**DOI:** 10.1038/s41377-023-01085-7

**Published:** 2023-03-03

**Authors:** Leanne Maurice, Alberto Bilenca

**Affiliations:** 1 Biomedical Engineering Department, Ben-Gurion University of the Negev, 1 Ben Gurion Blvd, Be’er-Sheva, 84105 Israel; 2 Ilse Katz Institute for Nanoscale Science and Technology, Ben-Gurion University of the Negev, 1 Ben Gurion Blvd, Be’er-Sheva, 84105 Israel

**Keywords:** Interference microscopy, Optical physics, Biophotonics

## Abstract

Single particle tracking in three dimensions is an indispensable tool for studying dynamic processes in various disciplines, including material sciences, physics, and biology, but often shows anisotropic three-dimensional spatial localization precision, which restricts the tracking precision, and/or a limited number of particles that can be tracked simultaneously over extended volumes. Here we developed an interferometric, three-dimensional fluorescence single particle tracking method based on conventional widefield excitation and temporal phase-shift interference of the emitted, high-aperture-angle, fluorescence wavefronts in a greatly simplified, free-running, triangle interferometer that enables tracking of multiple particles at the same time with <10-nm spatial localization precision in all three dimensions over extended volumes (~35 × 35 × 2 μm^3^) at video rate (25 Hz). We applied our method to characterize the microenvironment of living cells and up to ~40 μm deep in soft materials.

## Abstract (web summary)

Conventional widefield excitation and temporal phase-shift fluorescence interference in a basic 4π-interferometer enable video rate, multiple particle tracking with isotropic, 3D precision over large volumes in cells and deep in soft materials.

## Graphical abstract

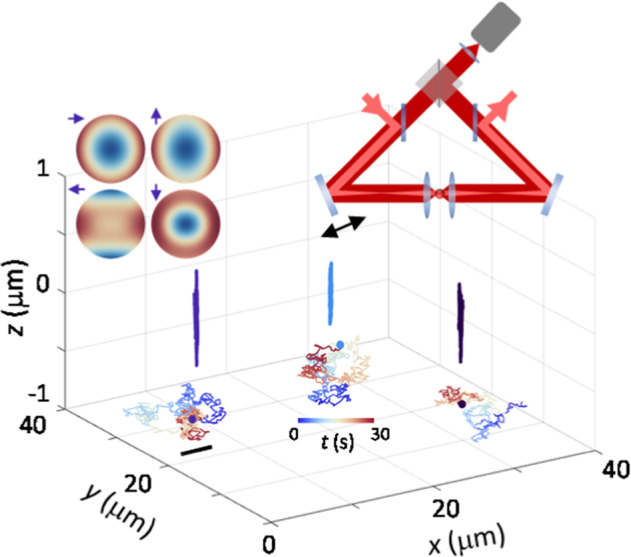

## Introduction

Single particle tracking (SPT) allows the direct measurement of particle motion in complex systems, such as gels and cells, providing important insights into their structure and function. While SPT across the lateral dimension is a relatively straightforward task, three-dimensional SPT (3D-SPT) with isotropic nanometer-level localization precision inside extended volumes remains a significant challenge. A long-standing method for 3D-SPT has been off-focus imaging^1–3^ that relies on the analysis of ring patterns in the defocused point spread function (PSF) images of the particle tracked. Although off-focus imaging can provide nanoscale localization precision that is comparable in all three directions at out-of-focus planes, it is not useful over the depth of field. Multifocal plane imaging^4–7^ overcomes this shortcoming by simultaneously acquiring images of the 3D-PSF at distinct (de)focus levels, yet it suffers from anisotropic 3D localization precision over the axial range. One class of methods that facilitates isotropic 3D localization precision in 3D-SPT is PSF engineering, including for example the double helix PSF^8,9^ which is simple to implement and widely used, though with the axial precision lower than the lateral one. Other methods in this class involve the use of tetrapod and corkscrew PSFs^10,11^. These methods have been used for 3D-SPT but are limited in the number of particles that can be tracked simultaneously due to the significantly increased extent of the engineered PSF on the camera. A different class of techniques for achieving isotropic 3D localization precision is based on 4π-steradian fluorescence self-interference—that is, the interference of the two opposing spherical wavefronts radiated from an isotropic fluorescent emitter. Among the methods in this class are 4Pi single marker switching microscopy (4Pi-SMS)^12–14^ and interferometric photoactivated localization microscopy (iPALM)^15–17^, which are often applied for superresolution imaging of biological nanostructures. Whereas these techniques have shown to achieve high isotropic 3D localization precision with micrometer-scale imaging depth using photoactivatable or photoswitchable fluorophores, they remain a tour de force due to the nontrivial spatial phase shifting setups with specialized components and active focus stabilization. Furthermore, they are not well suited for tracking multiple standard fluorescent particles simultaneously in three dimensions over extended volumes due to the limited number of particles that can be tracked over their restricted field of view and/or depth of field. Although 4Pi-SMS or iPALM might be used for high-density 3D-SPT of photoactivatable markers^18^, this would require specialized fluorophores and sample exposure to violet activation light, and the short trajectories recorded would not be useful for distinguishing between modes of motion or for extracting the motion speed. In addition, the diffusion coefficients may be fitted with limited statistical precision^18^. Interferometric methods for 3D-SPT of non-fluorescent scatterers also exist and include for example in-line holographic optical microscopy^19,20^ and computational imaging^21^.

Here, we report on temporal phase self-interferometry (TEMPSI), a video-rate three-dimensional single particle tracking (3D-SPT) method to simultaneously track multiple fluorescent beads with isotropic 3D localization precision (<10 nm) over extended volumes (~35 × 35 × 2 μm^3^) based on conventional widefield excitation and 4π-steradian fluorescence self-interference detection in a greatly simplified temporal phase shifting self-interferometry system comprising a compact free-running isosceles right triangle interferometer (hereafter referred to as 4π-cavity) with a phase-shifting mirror. Using TEMPSI, we characterized the microenvironment of homogeneous and inhomogeneous soft materials in 2-μm thick layers up to 40 μm deep inside the sample. Further, we applied TEMPSI for 3D-SPT in the microenvironment of living cells, illustrating its compatibility with biological systems.

## Results

**Fig. 1:**
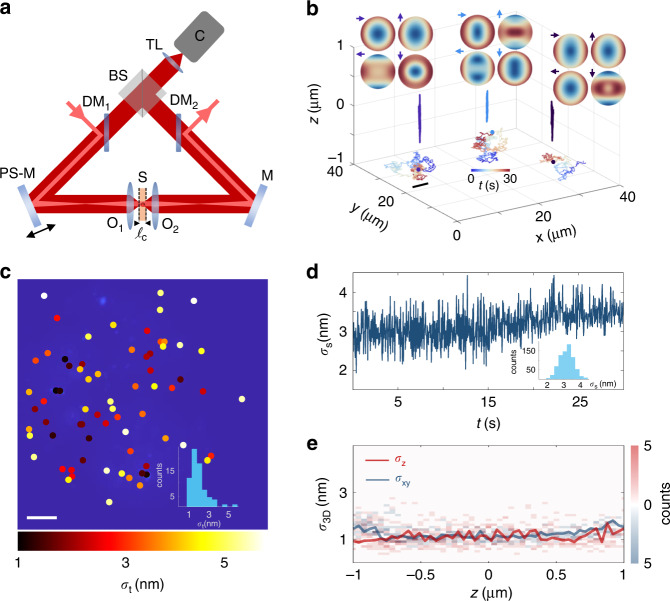
Method and characterization of TEMPSI. **a** In the TEMPSI system, the excitation beam (light red) is directed to the sample (S) via a dichroic mirror (DM_1_), the phase-shifting mirror (PS-M), and the objective lens (O_1_) to provide widefield illumination. The beam exists the system through the fixed mirror (M) and another dichroic mirror (DM_2_). The 4π-steradian fluorescence emitted around the zero optical path length difference point of the 4π-cavity (to within $\pm \ell _c/2$) is collected by the two opposing objectives lenses (O_1/2_) and the fluorescence self-interference at the beam splitter (BS) is imaged onto the camera (C) using a tube lens (TL). The phase-shifting mirror temporally changes the optical path length difference in the 4π-cavity, producing multiple interferograms from which the unambiguous nanometer localization of the fluorescent emitters is obtained in space. **b** 3D-SPT using TEMPSI is accomplished by acquiring four interferograms per 3D localization, with π/2 phase shift between consecutive frames (top insets where arrows signify the temporal phase shift introduced, explicitly 0, π/2, π, 3π/2). The continuous acquisition of the phase shift interferograms allows to 3D localize the particles across the field of view. The localizations are subsequently used to reconstruct the particle trajectories in space (shown in different colors with zoomed color-encoded time-elapsed insets). **c** Image of immobile fluorescent beads with the temporal standard deviation of their axial localizations *σ*_t_ in colored dots. The histogram of *σ*_t_ is also shown. Phase shift interferograms were acquired over 30 s at 100 Hz, resulting in 25 3D localizations s^−1^ with an average of ~60 × 10^3^ photons/localization. Scale bar, 5 μm. **d** Spatial standard deviation of the axial localizations *σ*_s_ as a function of time. The histogram of *σ*_s_ is also presented. *σ*_s_ was evaluated from the same data in (**c**). **e** TEMPSI localization precision in the lateral and the axial directions, *σ*_*xy*_ and *σ*_*z*_, over an axial range of 2 μm. The localization precision was evaluated using 10 immobile fluorescent beads scanned over an axial range of 2 μm. For each bead, we measured 50 localizations/axial-position at 25 Hz and with an average of ~130 × 10^3^ photons/localization. Color-coded histograms of the precision at each axial position are shown (*σ*_*xy*_, blue shades; *σ*_*z*_, red shades) along with the medians (solid lines with corresponding colors)

TEMPSI relies on the introduction of a rapidly time-varying phase shift *δ(t)* between the two opposing spherical wavefronts radiated from isotropic fluorescent emitters tracked near the midpoint of the base of a 4π-cavity to within $\pm \ell _c/2$, where $\ell _c$ is the coherence length of the fluorescence emission (Fig. 1a and Materials and methods). In general, *δ(t)* can be any desired phase shift waveform, where the sensitivity to errors in *δ(t)* typically decreases when more phase shifts are employed^22^. However, for 3D-SPT applications, it is desirable to reduce the motion-induced temporal modulation of the sequentially phase shifted interferograms by introducing less phase shifts. For simplicity, and following the four step spatial phase shifting algorithms used in 4Pi-SMS, we chose *δ(t)* to take four discrete values 0, π/2, π, and 3π/2 by repetitively steeping a phase-shifting mirror during 6 ms at 100 Hz repetition rate in increments of *λ*_e_/[8 × cos(*θ*)], where *λ*_e_ is the fluorescence emission wavelength and *θ* is the angle of incidence (92 nm for *λ* = 680 nm and *θ* = 22.5° used here). A time-varying phase shift PSF interferogram is then produced for each emitter (Fig. 1b), with the PSF and the relative phase between its two opposing spherical wavefronts encoded in these signals. TEMPSI obtains the unambiguous axial localization of the emitters from the inner and outer parts of the phase difference between these wavefronts using a lookup curve^12^ (Materials and methods and Fig. S1), and their lateral localization from the centroid of the intensity of the time-integrated PSF interferogram (Materials and methods). The emitter tracks are ultimately retrieved by connecting the 3D localizations acquired at 40 ms intervals (or 25 3D localizations s^−1^) with camera exposure time and frame rate of 4 ms and 100 Hz (Materials and methods).

Because TEMPSI obtains the axial localizations of an isotropic emitter from the phase difference between its opposing spherical wavefronts, we first characterized the spatiotemporal phase stability of the setup (Fig. 1c, d). Using multiple 170-nm fluorescent beads immobilized to a cover glass (Materials and methods), the temporal standard deviation of the axial localizations *σ*_t_ was measured for each bead across a ~35 × 35 μm^2^ field of view over 30 s (Fig. 1c), yielding a median value of 1.8 nm at 25 3D localizations s^−1^ with an average of ~60 × 10^3^ photons/localization. Further, we used the axial localizations measured to evaluate the spatial standard deviation *σ*_s_ across the field of view against time, resulting in a median value of 2.1 nm (Fig. 1d). These values of *σ*_t_ and *σ*_s_ indicate the high spatiotemporal phase stability of TEMPSI. Subsequently, we measured the 3D precision of TEMPSI over a 2-μm depth of field by translating a sample with multiple immobile fluorescent beads along the focal axis (Fig. 1e). 50 3D localizations/axial-position were recorded per bead at 25 3D localizations s^−1^ with an average of ~130 × 10^3^ photons/localization. Median values of 1–2 nm were achieved for the 3D localization precision over the entire depth of field. This result suggests that 3D localization precision better than 10 nm can be obtained for photon numbers higher than 10 × 10^3^ photons/localization assuming the localization precision is inversely proportional to the square root of the number of photons collected^15,23^ (Fig. S2).

**Fig. 2:**
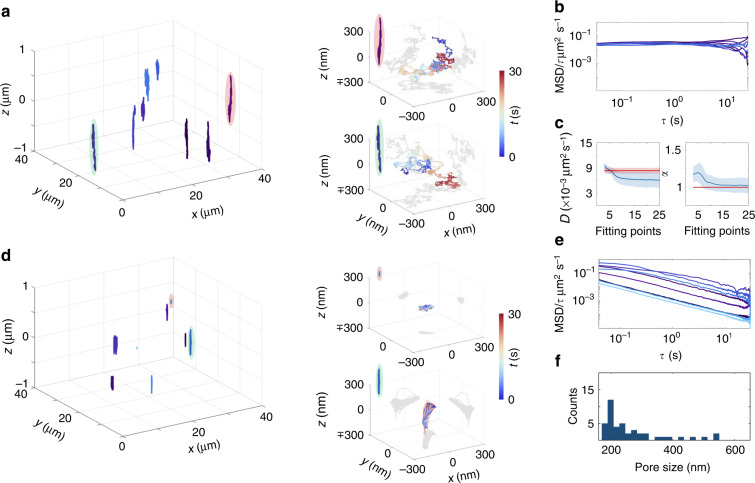
3D-SPT in homogeneous and heterogeneous soft materials by TEMPSI. **a** 3D trajectories of multiple fluorescent beads tracked simultaneously in a 90/10 glycerol/water mixture over 30 s at 25 3D localizations s^−1^ with an average of ~11 × 10^3^ photons/localization. Trajectories are presented in different colors. Zoomed trajectories of two beads marked by the green and red ovals in the left panel are also shown (right), with color reflecting time going from blue to red. **b** log–log MSD/τ curves of the trajectories in (**a**) coded with the same color of the corresponding trajectory. The vertical axis describes the ratio of the mean square displacement to the lag time. The horizontal straight lines are characteristic of purely diffusive motion in aqueous glycerol solutions. **c** Diffusion coefficient *D* and exponent *α* as a function of the number of fitting points (*n* = 231 trajectories). Blue solid lines and shaded regions represent mean and standard deviation values of the parameters fitted from the measured MSD curves, respectively. Red solid lines show the theoretical value of *D* at 20 °C and of *α* for a purely Brownian motion. The red shaded area in the left panel presents the theoretical values of *D* at 19–21 °C. **d** 3D trajectories of multiple fluorescent beads tracked simultaneously at ~40-μm depth in 1% agarose gel over 30 s at 25 3D localizations s^−1^ with an average of ~29 × 10^3^ photons/localization. Trajectories are presented in different colors (left). Zoomed trajectories of two beads marked by the green and red ovals in the left panel are also shown (right), where time is encoded as the color of the trajectory going from blue to red. The top trajectory shows confined motion in a pore small compared to the pore constraining the motion described by the bottom trajectory. **e** log–log MSD/τ curves of the trajectories in (**d**) coded with the same color of the corresponding trajectory. The vertical axis represents the ratio of the mean square displacement to the lag time. The negative slope lines are characteristic of constrained motion in agarose gels. **f** Histogram of the pore size of the agarose gel at ~40-μm depth (*n* = 46 trajectories)

To validate the effectiveness of TEMPSI for 3D-SPT, trajectories of multiple 170-nm diameter fluorescent beads were mapped simultaneously in 3D in a layer of 2 μm thickness within a glycerol/water solution (Materials and methods) over 30 s at 25 3D localizations s^−1^ with an average of ~11 × 10^3^ photons/localization (Fig. 2a). We used a glycerol/water ratio of 9:1 (v/v), balancing between correct tracking and rapid diffusion. By extracting the offset in the MSD curves of the 30-s-long trajectories measured in the solution (Materials and methods), the average 3D tracking precision was estimated to be 8.9 ± 3.7 nm. Together with the ~2.5-nm 3D localization precision at ~11 × 10^3^ photons (Fig. S2), it can be deduced that the tracking precision was dominated by the particle motion during the acquisition of the four interferograms rather than by the 3D localization precision due to photon noise^24^. The MSD curves exhibit horizontal straight lines on a log–log scale of the MSD/lag-time dependence (Fig. 2b). This temporal behavior of the MSD is characteristic of a purely diffusive motion, as expected for an aqueous glycerol solution. We further quantified the mean and standard deviation of the diffusion coefficient *D* and the exponent *α* of the motion as a function of the number of fitting points using the MSDs of the trajectories recorded over 30 s (Fig. 2c and Fig. S3a). Owing to the high tracking precision, the average *D* with minimum relative standard deviation was obtained using only 3 fitting points (*τ* = 40, 80, 120 ms) and yielded a value of 9.5 × 10^−3^ ± 7.7 × 10^−4^ μm^2^ s^−1^, which is consistent with the theoretical diffusion coefficient calculated from the Stokes–Einstein equation for the bead size used and the solution viscosity calculated at the lab temperature of 20 ± 1 °C (7.7 × 10^−3^– 9.05 × 10^−3^ μm^2^ s^−1^ as marked by the red region in the left panel of Fig. 2c). The average *α* with minimum relative standard deviation was estimated using 14 fitting points (*τ* = 40, 80,…, 560 ms) and resulted in a value of 1.03 ± 0.09, which agrees well with the expected *α* = 1 of a purely diffusive motion (red line in the right panel of Fig. 2c). The number of fitting points required to minimize the relative standard deviation in *α* was larger than for *D* because the exponent *α* dominates at later lag times than diffusion. Consequently, the estimation of *α* required trajectories longer than 18 s for relative standard deviations smaller than 10%, whereas even trajectories as short as ~0.5 s were sufficient in estimating *D* with <10% relative standard deviations (Fig. S3b).

To study TEMPSI capabilities for 3D-SPT in more complex media, we chose to track 170-nm diameter fluorescent beads in a layer of 2 μm thickness within a 120-μm thick 1% agarose gel (Materials and methods), which displays spatial inhomogeneity and many small pores. The trajectories of multiple beads were recorded simultaneously at a ~40-μm depth over 30 s at 25 3D localizations/s^−1^ with an average of ~29 × 10^3^ photons/localization (Fig. 2d). The 3D tracking precision was assessed to be 14.7 ± 8.7 nm from the offset in the MSDs. As the 3D localization precision at ~29 × 10^3^ photons is ~2 nm (Fig. S2), we can conclude that the tracking precision was again dominated by the motion of the particles during the multiple interferogram acquisitions. The MSD curves of the trajectories revealed a linear behavior with negative slopes on a log-log scale of the MSD/lag-time dependence (Fig. 2e), characteristic of constrained motion which is expected for agarose gels. We further retrieved the pore size distribution at that depth from the plateau of the MSDs averaged over *τ* = 1−25 s^25^ and with correction for the offset in the MSD curves (Fig. 2f and Materials and methods). From the distribution measured, we observe that the measured pore size varied in the range ~180–550 nm with an average pore size of 265 ± 100 nm, agreeing well with other methods^26–28^. Pores smaller than 170 nm could not be measured because of the particle size used here. In addition, pores larger than 550 nm were not identified as in one previous report^28^ likely due to differences in the materials and methods used to prepare the agarose^27^. As a further control, we repeated the measurements and analysis at a depth of ~10 μm within the gel and obtained similar results (Fig. S4).

**Fig. 3:**
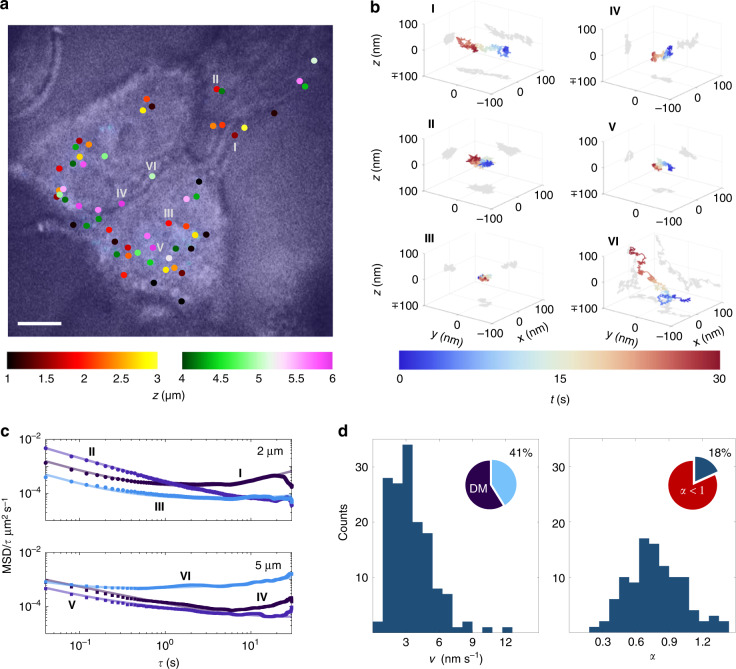
3D-SPT in human lung carcinoma (A549) cells by TEMPSI. **a** Overlay of white light and fluorescence images of representative A549 cells with embedded beads. Six beads whose tracks are shown in (**b**) are numbered in Roman numerals. Color reflects imaging depths of ~2-μm and ~5-μm relative to the glass-cell interface (hot and green-pink colormaps, respectively). The beads at each depth were tracked simultaneously over 30 s at 25 3D localizations s^−1^ with an average of ~35 × 10^3^ photons/localization. Scale bar, 5 μm. **b** 3D trajectories of the six beads numbered in Roman numerals in (**a**), where the trajectory color encodes time going from blue to red. **c** log–log MSD/τ curves of the trajectories in (**b**). The vertical axis shows the ratio of the mean square displacement to the lag time. Dots and squares are measurements and solid lines are fits. The curved convex lines (I and IV), the negative slope lines (II and V), and the slow slope curves straightened at longer lag times (III and VI) are characteristic of directed, subdiffusive, and diffusive motions in the cell microenvironment, respectively. **d** Histograms of the speed (*v*, left; *n* = 149 trajectories) and the exponent *α* (right; *n* = 104 trajectories) of the motion in the cell microenvironment. 59% of the trajectories showed directed motion (DM), whereas from the remaining 41% trajectories, 82% exhibited *α* < 1

We next demonstrate the compatibility of TEMPSI with biological systems by imaging the 3D tracks of fluorescent beads in the surface and/or internal microenvironment of living human lung carcinoma (A549) cells (Materials and methods), which are widely used as an in vitro model in lung cancer research. Multiple 3D trajectories were mapped simultaneously inside a layer of 2 μm thickness, distant ~2 μm and ~5 μm from the glass-cell interface, over 30 s at 25 3D localizations s^−1^ with an average of ~35 × 10^3^ photons/localization (Fig. 3a, b). The 3D tracking precision estimated from the offset in the MSDs yielded a value of 3.9 ± 1.8 nm—still overriding the 3D localization precision (~1.5 nm at ~35 × 10^3^ photons as observed in Fig. S2). To characterize the motion of the beads in the microenvironment of the cells, we analyzed their MSDs against lag time and for simplicity classified the motion as directed, if a component of quadratic dependence of the MSD on lag time was identified (on a linear scale), and as non-directed otherwise (Fig. 3c and Materials and methods). Using 150 fitting points (*τ* = 0.04, 0.08, …, 6 s) and the 30-s-long trajectories, the relative standard deviations in the speed and the exponent *α* measurements were optimized. Because of the large, so-called, reduced localization error—the ratio of the squared tracking precision to the squared displacement of the particle— in the cells, many points were required in the fits, and as such the relative variation in the estimate of the motion parameters increased^24^. We found that 59% out of 253 trajectories exhibited a slow directed motion with an average (median) speed of 3.5 ± 1.9 nm s^-1^ (3.1 nm s^−1^) (Fig. 3d, left), which may be associated with active motion inside the cells^29^ or to dynamic interaction with the cell membrane^30^. The remaining 41% of the trajectories presented diffusive motion, where 82% of these trajectories were subdiffusive with *α* < 1 and an average (median) value of 0.78 ± 0.24 (0.76) (Fig. 3d, right), possibly due to diffusion affected by the microenvironment in the vicinity of the moving particles^29–31^ (termed as passive or confined motion). Further, irrespective of whether the beads were within the cell or on its surface, their dynamics was significantly different from that observed for immobile beads (Fig. S5). Certainly, our results are in reasonable agreement with the results of particle tracking in other living cells, considering the difference in the particle size. For instance, the MSD analysis of the tracks of 35-nm and 70-nm fluorescent nanodiamond moving in HeLa cells, resulted in speeds of ~8 nm s^-1^ and ~2 nm s^−1^ and *α* smaller than ~0.8^32,33^. Another example is the MSD analysis of the trajectories of 100-nm fluorescent beads in 3T3 cells that yielded speeds of ~6 nm s^−1^ and *α* smaller than 1^34^.

## Discussion

In this work, we developed TEMPSI and showed its strength in robust 3D-SPT of fluorescent beads inside large volumes. Factors to be considered to reliably estimate the diffusion coefficient and the mode of motion are (i) the signal-to-noise-dependent tracking uncertainty which decreases with the total number of photons collected, (ii) the washout of the interferogram visibility due to particle motion during the camera exposure time, and (iii) the temporal, intensity and phase, modulation of the interferograms because of particle motion during the camera exposure time and the mirror stepping time. All these factors need to be minimized. In addition, the MSD analysis requires that trajectories of several hundreds of spatial coordinates be acquired and that the number of fitting point be optimized for achieving adequate statistical precision in the estimate of the motion parameters^24^. Whereas we used TEMPSI with bright fluorescent beads (>10,000 photons/localization), the application of dimmer emitters would decrease the effective depth of field and the localization precision (and, hence, possibly the tracking precision) due to the lower signal-to-noise ratio. Consequently, the reduced localization error would increase, decreasing the statistical precision in the estimation of the motion parameters^24^. For example, the use of 1,000 photons/localization instead of 10,000 photons/localization in the 90/10% glycerol/water mixture would reduce the localization precision by a factor of (10,000/1,000)^½^ ~3 in the photon noise limit, somewhat increasing the relative statistical error in the fitted motion parameters^24^. This could be alleviated by using both ports of the 4π-cavity beam splitter for doubling the number of photons collected and/or by means of improved SPT analyses. To minimize the washout of the interferogram visibility and the temporal modulation of the interferograms induced by the particle motion, the camera exposure time was set to the minimum that permitted >10,000 photons to be collected per localization (4 ms), while the mirror stepping time was set to the minimum allowable by the piezo nanopositioner (6 ms). The use of the two 4π-cavity ports for the instantaneous measurement of a pair of π-shifted interferograms with 0 and π/2 shifts introduced by the phase-shifting mirror would increase the acquisition rate of the four interferograms by twofold, further minimizing motion artifacts which may be advantageous for 3D-SPT of faster dynamic processes. Other approaches for tracking rapid dynamics include replacing the existing mirror nanopositioner with a faster one and/or shortening the camera exposure time using brighter particles, such as quantum dot beads. Another aspect to consider when using TEMPSI is the coherence length of the fluorescent emitters, which could limit the useful depth of field. The use of more narrowband fluorescent emitters, such as fluorescent nanodiamonds, may therefore be useful. As a last point, the length of the trajectories mapped here (30 s) was limited by photobleaching of the fluorescent beads and by the video recording system. Better video recorders and reduced photobleaching, for example using antifading reagents or more photostable particles, including fluorescent nanodiamonds and quantum dot beads, would enable, together with active focus stabilization, the acquisition of longer tracks.

With its high isotropic 3D localization precision and large volume of field, TEMPSI offers an effective means for the simultaneous, 3D tracking of multiple particles in soft materials and cells at video rate. As TEMPSI works best with strong and narrowband fluorescence, the use of bright fluorescent emitters having sufficiently narrow emission band is essential. Together with its more accessible setup, TEMPSI-based 3D-SPT may open new possibilities for studying complex dynamics in the 3D microenvironment of physical and biological systems.

## Materials and methods

### TEMPSI setup

The TEMPSI system is illustrated in Fig. 1a. The collimated excitation laser (633 nm; Melles Griot) enters a compact isosceles right triangle interferometer (hypotenuse length of ~350 mm) via a dichroic mirror (DM_1_; Chroma) and is then focused to the back aperture of the objective lens (O_1_; 1.15 × 63, Zeiss) to illuminate a wide area of the sample (~40-μm in diameter). A second identical dichroic mirror (DM_2_) is used to remove the excitation beam out of the 4π-cavity and to balance dispersion. While fluctuations in the incidence angle or transverse offset of the collimated laser beam tilt or resize the widefield excitation beam on the sample, the fluorescence emission of the beads excited in the sample is nearly isotropic (as the fluorescent molecules constituting them are randomly oriented), thus phase instabilities in the fluorescence self-interference patterns due to the excitation laser pointing fluctuations are negligible. The sample is sandwiched between two cover glasses and is mounted on a three-axis nanopositioner (Mad City Labs and Newport) near the midpoint of the base of the 4π-cavity to within $\pm \ell _c/2$, where $\ell _c$ is the coherence length of the fluorescence emission. Two identical water-immersion objective lenses are used to collect the fluorescent light emitted over a 4π solid angle. The fluorescent beams are then directed through the two mirrors of the 4π-cavity (PS-M and M; Thorlabs) to a neutral beam splitter (BS; Thorlabs), where they interfere upon being recombined. The resulting interference pattern is imaged using a 250-mm tube lens (Thorlabs) on a sCMOS camera (C; Hamamatsu) with an emission filter (Semrock).

The difference in the pathlength between the two arms of the 4π-cavity is introduced by translating a phase-shifting mirror (PS-M) at nonnormal incidence (~22.5°) using a one-axis nanopositioner (Mad City Labs). Ultimately, this incidence angle together with a similar incidence angle on the fixed mirror in the 4π-cavity necessitate to be as accurate as to obtain visible fluorescence self-interference fringes on the camera using immobile fluorescent beads (Supplementary Manual). Because of the nonnormal incidence, a small lateral displacement is produced, but it is negligible since the light field is collimated at the back aperture of the objective lens. The phase-shifting mirror and the camera are synchronized using a LabVIEW FPGA Module (NI), where first the mirror is shifted to its location and then the camera is exposed. The images are saved via StreamPix on a data acquisition computer at 100 Hz. The alignment procedure of the TEMPSI system is described in the Supplementary Manual.

### Determination of the axial localization

In TEMPSI, a four step phase shifting algorithm obtains the axial localization of a fluorescent emitter by recording four interferograms^35^ *I*_*k*_(*ρ*,*ϕ;z*_o_), *k* = 1, 2, 3, 4

1 $$I^\prime \left( {\rho ,\phi ;z_o} \right) + I{^{\prime\prime}}\left( {\rho ,\phi ;z_o} \right){{{\mathrm{cos}}}}\left[ {\Delta \varphi \left( {\rho ,\phi ;z_{{{\mathrm{o}}}}} \right) - \delta _k} \right]$$

where *δ*_1_ = 0, *δ*_2_ = π/2, *δ*_3_ = π, *δ*_4_ = 3π/2, the coordinates *ρ* and *ϕ* are in the camera plane, and *z*_o_ is the axial location of the emitter in the object space relative to the zero optical pathlength difference point of the 4π-cavity which coincides with the joint focus of the two objective lenses. Also, $I^\prime$ represents the sum of the intensity PSFs of the two objective lenses, $I{^{\prime\prime}}$ stands for the product of the moduli of their amplitude PSFs, and Δ*φ* denotes the phase difference between them.

To obtain the inner and outer parts of Δ*φ*, Δ*φ*^in/out^(*z*_o_), from which TEMPSI retrieves the unambiguous axial localization of an emitter, the Gaussian-weighted zeroth and third moments $\bar I_k^{{{{\mathrm{in}}}}/{{{\mathrm{out}}}}}(z_{{{\mathrm{o}}}})$ of *I*_*k*_(*ρ*,*ϕ;z*_o_) are first computed as^12,35^

2 $$\mathop {\int}\limits_0^{2\pi } {\mathop {\int}\limits_0^\infty {\rho ^nG(\rho )I_k\left( {\rho ,\phi ;z_{{{\mathrm{o}}}}} \right)\rho {\rm{d}}\rho {\rm{d}}\phi } }$$

where *n* = 0, 3 for the zeroth and third moments and *G* is a Gaussian mask with zero mean and variance *σ*^2^. Δ*φ*^in/out^(*z*_o_) is next calculated as^35^

3 $${{\Delta }}\varphi ^{{{{\mathrm{in}}}}/{{{\mathrm{out}}}}}(z_{{{\mathrm{o}}}}) = - {{{\mathrm{tan}}}}^{ - 1}\left[ {\frac{{\bar I_4^{{{{\mathrm{in}}}}/{{{\mathrm{out}}}}}(z_{{{\mathrm{o}}}}) - \bar I_2^{{{{\mathrm{in}}}}/{{{\mathrm{out}}}}}(z_{{{\mathrm{o}}}})}}{{\bar I_1^{{{{\mathrm{in}}}}/{{{\mathrm{out}}}}}(z_{{{\mathrm{o}}}}) - \bar I_3^{{{{\mathrm{in}}}}/{{{\mathrm{out}}}}}(z_{{{\mathrm{o}}}})}}} \right]$$

The axial localization of the emitter *z*_o_ is finally determined by unwrapping Δ*φ*^in^(*z*_o_) using Δ*φ*^out^(*z*_o_) in the lookup curve (see below) and relating the unwrapped Δ*φ*^in^(*z*_o_) to *z*_o_ via the proportionality constant *λ*_eff_/4π with *λ*_eff_ being the effective wavelength under high numerical aperture focusing^36,37^ or through the position readings of the sample nanopositioning system.

### (Δφ^in^,Δφ^out^) lookup curve

To measure the lookup curve, we translated a sample with immobile fluorescent beads (see below) along the focal axis over 2 μm and recorded 5 axial localizations/position at 11.9 axial-localizations s^−1^ with a 15-ms camera exposure time. The mean of the 5 measurements was defined as a single [Δ*φ*^in^(*z*_o_), Δ*φ*^out^(*z*_o_)] point of the lookup curve. The measured points of the lookup curve were subsequently fiftyfold interpolated (solid line in Fig. S1 where the color encodes *z*_o_). To demonstrate unambiguous axial localizations using the lookup curve, another sample with immobile fluorescent beads was scanned over a 2-μm depth of field using 50 axial-localizations/position acquired at 25 axial-localizations s^−1^ with a camera exposure time of 4 ms. The axial localizations were achieved by mapping the measured values of Δ*φ*^in^ and Δ*φ*^out^ to the nearest point on the lookup curve and then assigning to this point the corresponding *z*_o_ value. Distinguishable clusters of axial localizations are clearly observed on the lookup curve over the entire depth of field of 2 μm (dots in Fig. S1).

### Determination of the lateral localization

In TEMPSI, the sum of the four interferograms measured $\mathop {\sum}\nolimits_{k = 1}^4 {I_k\left( {\rho ,\phi ;z_{{{\mathrm{o}}}}} \right)}$ yields the sum of the intensity PSFs of the two objective lenses $I^\prime \left( {\rho ,\phi ;z_o} \right)$. Thus, the lateral localization is obtained by nonlinear least square fitting of $I^\prime \left( {\rho ,\phi ;z_o} \right)$ to a Gaussian model.

### TEMPSI 3D-SPT algorithm

The 3D coordinates of a trajectory were determined as described above using a lookup curve measured prior to the tracking experiments. Phase measurements were corrected for systematic errors using the phase values measured for the zero optical pathlength difference point of the 4π-cavity. Mislocalizations in the trajectories were corrected using the surrounding axial estimates^35^. Sample drift was corrected by calculating the collective motion of all particles tracked in the frame and subtracting it from each of the individual trajectories^38^.

### Mean square displacement (MSD) analysis of single bead trajectories

The MSD curve is expressed as^24,39^

4 $$\left\langle {\left\| {{{\Delta }}\bar r} \right\|^2(n)} \right\rangle = \frac{1}{{N - n}}\mathop {\sum }\limits_{i = 1}^{N - n} \left\| {\bar r_{i + n} - \bar r_i} \right\|^2,\;n = 1,2, \ldots ,N - 1$$

where $\bar r_i = \left( {x_i,y_i,z_i} \right),\;i = 1,\;2, \ldots ,\;N$ is the *i*-th 3D coordinate of the trajectory and *n* is the lag index which is related to the lag time *τ* via *τ* = *n* × Δ*t* with Δ*t* being the time interval between successive 3D localizations.

The motion modes of the beads in all the samples were analyzed by nonlinear least square fitting of the MSD curves to the general model *D*_α_ × *τ*^*α*^ + *v*^2^*τ*^2^ + *C*, where *D*_α_ is the generalized diffusion coefficient, *α* represents the exponent parameter of the motion (0 < *α* *<* 1, subdiffusion; *α* = 1, pure diffusion; *α>*1, superdiffusion), *v* is the speed of the directed motion, and *C* is an offset related to the localization and tracking uncertainties and the camera exposure time^24,39^. The offset was estimated using the number of fitting points that yields the minimum relative standard deviation. Classification between directed and non-directed motion was based on the residual standard error, the coefficient of determination of the fits, and the dominant component of the motion.

To estimate the pore size in the agarose gel samples, we used the plateau of individual MSD curves and approximated the pore size as $a + \sqrt {\left\langle {\left\| {{{\Delta }}\bar r} \right\|^2(\infty )} \right\rangle }$ where *a* is the bead size and $\sqrt {\left\langle {\left\| {{{\Delta }}\bar r} \right\|^2(\infty )} \right\rangle }$ is the size of the gap between the bead surface and the wall of the pore^25^. $\left\langle {\left\| {{{\Delta }}\bar r} \right\|^2(\infty )} \right\rangle$ was corrected for the localization and tracking uncertainties by subtracting from it the offset *C* described above.

### Preparation of the immobile fluorescent beads sample

50 μL Poly-L-Lysine (Sigma-Aldrich) was dried on a 25-mm diameter #1 cover glass. The cover glass was then washed with double distilled water (DDW) and blow dried with air. 170-nm fluorescent beads (Invitrogen 660/680) were next diluted 1:150 with DDW. 10 μL of the beads were dried on top of the Poly-L-Lysine layer, washed with DDW, blow dried with air, and covered by a second clean cover glass.

### Preparation of the glycerol/water samples

170-nm fluorescent beads (Invitrogen 660/680) were mixed in a 90/10% glycerol/water solution at a ratio of 2.68 × 10^9^ particles ml^−1^. A vortex was used to obtain a homogeneous sample mixture. For the tracking experiments, the mixture was sandwiched between two 25-mm diameter #1 cover glasses.

### Preparation of the agarose gel samples

0.7 mM phosphate buffered saline (PBS) was mixed with triton ×100 in a final concentration of 0.02% (v/v). Agarose powder (Sigma-Aldrich) 1% (w/v) was then added and the mixture was placed in a thermo-shaker for ~10 min at 90 °C and 200 rpm. 170-nm fluorescent beads (Invitrogen 660/680) were added to the mixture at a ratio of 2.68 × 10^9^ particles/ml prior to gelation. A vortex was used to obtain a relatively homogeneous sample mixture. For the tracking experiments, the mixture was sandwiched between two 25-mm diameter #1 cover glasses with a 120-μm thick spacer (Grace Bio-Labs). Beads were also immobilized to the cover glass to serve as a reference plane for the tracking depth assessment.

### Preparation of the A549 cell samples

A549 cell line, originally derived from a human lung carcinoma (ATCC^®^ CCL-185™), was used. The cells were grown in Dulbecco’s-modified eagle’s medium (DMEM), supplemented with 100 U ml^-1^ penicillin and 100 μg ml^−1^ streptomycin (1% Pen-Strep), 2 mM L-glutamine and 10% fetal bovine serum. The cells were maintained in a humidified atmosphere of 5% CO_2_ at 37 °C. 24–48 h prior to the tracking experiments, 6 ml of cells (4 × 10^5^ cells ml^−1^) were transferred into a tissue culture (TC) plate containing cover glasses with immobile 170-nm fluorescent beads (Invitrogen 660/680). Cells were plated on a cover glass to which beads were immobilized to enable the identification of the glass-cell interface, which served as a reference plane for estimating the tracking depth. Immediately before the experiments, one cover glass was moved to a new TC plate and was covered with 1.5 ml of DMEM mixed with the fluorescent beads (5.9 × 10^10^ particles ml^−1^). The cells were exposed for 30 min to the beads in the incubator, providing a suitable concentration of particles for robust particle tracking measurements in the cells. The cover glass was next washed three times with PBS to reduce particle adsorption to the cell surface. Finally, a 120-μm thick spacer (Grace Bio-Labs) was glued to a second 25 mm diameter cover glass, 20 μL of PBS were added to it, and the two cover glasses were attached together and mounted for imaging by the TEMPSI system using Viscotears (Ophthalmic gel, Novartis) as the objective immersion medium.

## Supplementary information

Supplementary Information

Supplementary Manual
