# Supplementary material for: Three-dimensional single particle tracking using 4π self-interference of temporally phase-shifted fluorescence
Source: Light Sci Appl. 2023 Mar 3;12:58. doi: 10.1038/s41377-023-01085-7 (PMC9981587; doi:10.1038/s41377-023-01085-7)
Supplement: Supplementary file 1 — Supplementary Information [file 41377_2023_1085_MOESM1_ESM.docx]

Supplementary Information

**Three-dimensional single particle tracking using 4π self-interference of temporally phase-shifted fluorescence**

Leanne Maurice^1^ & Alberto Bilenca^1,2*^

^1^Biomedical Engineering Department, Ben-Gurion University of the Negev, 1 Ben Gurion Blvd, Be’er-Sheva 84105, Israel

^2^Ilse Katz Institute for Nanoscale Science and Technology, Ben-Gurion University of the Negev, 1 Ben Gurion Blvd, Be’er-Sheva 84105, Israel

e-mail: *bilenca@bgu.ac.il

**Fig. S1** │ Lookup curve that relates the axial localization of a fluorescent bead, *z*_o_, to the measured inner and outer parts of the phase difference between the two opposing spherical wavefronts radiated from the bead, Δ*ϕ*^in^ and Δ*ϕ*^out^ (solid lines). Clusters of axial localizations of lower signal-to-noise-ratio (SNR) beads are also shown (dots). Color encodes *z*_o_. The lookup curve was obtained by scanning a fluorescent bead immobilized to a cover glass over 2 μm with 5 axial-localizations/position at 11.9 axial-localizations/s and 15-ms camera exposure time. The mean of the 5 measurements determines a single [Δ*ϕ*^in^(*z*_o_), Δ*ϕ*^out^(*z*_o_)] point of the lookup curve. The measured points were subsequently fiftyfold interpolated. Clusters of axial localizations were measured by scanning a lower SNR immobile fluorescent bead over 2 μm with 50 axial-localizations/position at 25 axial-localizations/s and a 4-ms camera exposure time. The axial localization was obtained by locating the point on the lookup curve that minimizes the Euclidean distance between the phase pair [Δ*ϕ*^in^, Δ*ϕ*^out^] measured and the lookup curve.

**Fig. S2** │ 3D localization precision, *σ*_3D_, of immobile fluorescent beads at the focus as a function of the photon number detected, *N*. *σ*_3D_ was determined by TEMPSI using 25 beads at 25 3D-localizations/s with a 4-ms camera exposure time. Middle and length of error bars are mean and variability (in standard deviation), respectively. Solid lines are fits to the root square of a biased hyperbola^12^.

**Fig. S3** │ **Statistical analysis of MSD curves of glycerol/water mixtures.** **a,** The relative standard deviation of the diffusion coefficient *D* (blue line) and the exponent *α* (gray line) as a function of the number of fitting points. *D* and *α* were extracted from nonlinear fits to the MSD curves of 30-s-long trajectories of 231 beads (Material and methods). **b,** *D* and *α* as a function of the trajectory length (*n*=231 trajectories). The number of fitting point for *D* and *α* was 3 and 14, respectively. Blue solid lines and shaded regions represent mean and standard deviation values of the parameters fitted from the MSD curves of the trajectories segmented to the desired temporal length, respectively. The insets show the relative standard deviation (RSD) of *D* and *α* against the trajectory length.

**Fig. S4** │ **3D-SPT in 1% agarose gel at** **~10-μm depth. a,** 3D trajectories of multiple fluorescent beads tracked simultaneously over 30 s at 25 3D-localizations/s with an average of ~28×10^3^ photons/localization. Trajectories are presented in different colors (left). Zoomed trajectories of two beads marked by green and red ovals in the left panel are also shown (right), where time is encoded as the color of the trajectory going from blue to red. The top trajectory shows confined motion in a pore small compared to the pore constraining the motion described by the bottom trajectory. **b,** log-log MSD curves of the trajectories in **a** coded with the same color of the corresponding trajectory. The vertical axis represents the ratio of the mean square displacement to the lag time. The negative slope lines are characteristic of constrained motion in agarose gels. **c**, Histogram of the pore size of the gel at ~10-μm depth (*n*=90 trajectories). The mean pore size was 261±90 nm.

**Fig. S5** │ **Characterization of immobile and moving fluorescent beads in the A549 cell samples.** **a,** Overlay of white light and fluorescence images of a representative A549 cell sample with beads immobilized to the sample cover glass (left) and beads embedded at ~2-μm depth (right). Scale bar, 5 μm. **b,** log-log MSD curves of the immobile (red; *n*=168 trajectories) and moving (blue; *n*=253 trajectories) beads in the sample. Trajectory data was acquired as in Fig. 3. The vertical axis represents the ratio of the mean square displacement to the lag time. The solid lines are median values, and the bottom and top outlines of the shaded areas are the 25th and 75th percentiles of the MSDs. The MSDs reveal that the immobile beads had a median displacement of ~10 nm in 30 sec, attributed to the slow drift of the system, whereas the beads in the cells experienced ~5-6-fold larger displacements. Two-sample *t*-tests at each lag time confirmed that the motion of the beads varied significantly between the immobile and moving bead groups at the 5% significance level.
