# Supplementary material for: Three-dimensional single particle tracking using 4π self-interference of temporally phase-shifted fluorescence
Source: Light Sci Appl. 2023 Mar 3;12:58. doi: 10.1038/s41377-023-01085-7 (PMC9981587; doi:10.1038/s41377-023-01085-7)
Supplement: Supplementary file 2 — Supplementary Manual [file 41377_2023_1085_MOESM2_ESM.docx]

Supplementary Manual

**Three-dimensional single particle tracking using 4π self-interference of temporally phase-shifted fluorescence**

Leanne Maurice^1^ & Alberto Bilenca^1,2*^

^1^Biomedical Engineering Department, Ben-Gurion University of the Negev, 1 Ben Gurion Blvd, Be’er-Sheva 84105, Israel

^2^Ilse Katz Institute for Nanoscale Science and Technology, Ben-Gurion University of the Negev, 1 Ben Gurion Blvd, Be’er-Sheva 84105, Israel

e-mail: *bilenca@bgu.ac.il

**Basic setup**

The key optical components of the TEMPSI system are mounted on commercially available mounts with the following degrees of freedom (Fig. 1a in the main text):

1. BS (BS013, Thorlabs) is mounted on a 3-axis (pitch, roll, yaw) stage (PO46BL-50, Newport)

2. PS-M (BB1-E02, Thorlabs) is mounted on a 1-axis translation stage with pitch and yaw adjustments (NANO-OP30, Mad City Labs placed on a 420 linear stage, Newport)

3. O_1_ (421887-9970-000, Zeiss) is mounted on a 5-axis (x, y, z, pitch, and yaw) stage with a custom-made adapter (Nano-LPQ, Mad City Labs; POLARIS-K1T2, Polaris)

4. S is placed in a custom-made holder with pitch and yaw adjustments mounted on a 3-axis translation stage (MicroStage, Mad City Lab; Picomotor, Newport)

5. O_2_ (421887-9970-000, Zeiss) is mounted on a 5-axis (x, y, z, pitch, and yaw) stage (LP-1A, Newport)

6. M (BB1-E02, Thorlabs) is mounted on a 1-axis translation stage with pitch and yaw adjustments (PT1A ,Thorlabs; POLARIS-K1-H, Polaris)

7. TL (AC254-250-A, Thorlabs) and C (ORCA- flash 4.0 V2) are mounted on an optical rail (Newport)

General guidelines for constructing the TEMPSI system are:

1. To facilitate the construction of the system, use optical rails for aligning the key components (i.e, BS, PS-M, M, TL, and C)

2. To ensure the base angles of the 4π-cavity are as close as possible to 45°, we used a CNC-machined isosceles right triangle between the base and leg rails of the cavity

3. The visibility of the fringes obtained with either white light or immobile beads should be at least 0.5. Otherwise, the system must be realigned

4. The excitation laser, optical filters, and dichroic mirrors should be selected according to the excitation and emission wavelengths of the fluorescent particles employed in the particle tracking experiments

**Alignment procedure**

The comprehensive alignment of the TEMPSI system consists of the following 3 steps:

I. Alignment of the 4π-cavity

II. Alignment of the excitation laser and optics

III. Alignment of the camera and imaging optics

Note that maintenance of the system is obtained by a routine alignment procedure prior to the measurement and will be described at the end of this section. It worth noting that all beams used should be at the same height above the optical table.

*I. Alignment of the 4π-cavity*

The coarse alignment of the 4π-cavity starts using a collimated continuous wave laser beam entering the cavity through BS, whereas the finer alignment employs a collimated white light beam along with a double-sided mirror mounted on the sample holder. The double-sided mirror comprises a 25-mm diameter #1 glass coverslip where half of its facet is coated with a 100±5 nm layer of Ag and a 20±5 nm layer of SiO2, and another glass coverslip on top of the coated one. Double distilled water is used as the medium between the two coverslips.

1. Use the continuous wave laser beam for illuminating BS. Note step 1 in *Alignment of the excitation optics*

2. Place BS and align its tilt and rotation such that the beams exiting BS are parallel to the optical table

3. Place PS-M and align the reflected beam to create a ~45° with the beam emerging from BS. Note step 2 in *Alignment of the excitation optics*

4. Place the second mirror M. This mirror should be positioned a little farther away from the desired final location in the cavity. Note that before placing this mirror, it is recommended to place all the optomechanical components between M and PS-M (not including the two opposing objectives)

5. Align the tilts of M to obtain two counter-propagating beams travelling parallel to the optical table inside the cavity

6. Slowly move M towards PS-M. Both beams that exit BS should come closer to each other. When the beams overlap, an interference pattern should be visible behind BS. Stop moving M when both beams overlap and the interference pattern is dark. To improve the quality of the interference pattern, adjust the tilts of M. For better alignment of the tilts of M use the white light beam for illuminating BS

7. Place the double-sided mirror in the sample holder

8. Using an auxiliary lens in front of BS, focus the laser beam on the double-sided mirror. Confirm that the laser is roughly focused onto the same spot on both sides of the double-sided mirror. Otherwise, realign the cavity using steps 1-6

9. Tilt the sample holder to overlay the laser beams at the output of BS

10. Locate the zero optical pathlength difference point of the 4π-cavity. To this end, first observe circular fringes of the laser at the output of BS. Next, move the double-sided mirror sample forward and backward to identify the direction in which the fringes move inwards

11. Use the white light beam to finely locate the zero optical pathlength difference point of the cavity by scanning the double-sided mirror sample towards the direction determined in step 10 until white light fringes are visible. Calculate the fringe visibility. For values smaller than 0.5, move M forward or backward, adjust the mirror tilt, and observe for white light fringes again, until a fringe visibility of at least 0.5 is obtained

12. Adjust the tilt of O_1_ and O_2_. To this end, first place two auxiliary mirrors on the objective mounts with their reflecting surfaces pointing in opposite directions towards BS. Next, adjust the tilts of the objective mounts to obtain laser beams that are aligned at the exit of BS. Remove the auxiliary mirrors and place O_1_ and O_2_ in their mounts.

13. Place a drop of Viscotears (ophthalmic gel, Novartis) or another immersion medium on O_1_. Adjust the x-y position of O_1_ until the laser beam is aligned at the exit of BS

14. Place O_2_ in the cavity by repeating step 13

15. Use the white light source and an auxiliary iris in front of BS to adjust the axial positions of O_1_, O_2_, and TL by imaging the iris on C

16. Confirm that a fairly sharp white light fringe is still observed on C by translating PS-M. If necessary, move the double-sided mirror sample and the two objectives together to obtain a fairly sharp fringe

*II. Alignment of the excitation laser and optics*

For excitation, a laser is focused on the back aperture of O_1_. Dichroic mirrors DM_1_ and DM_2_ are used to direct the excitation beam to the sample and then to remove it out of the 4π-cavity. DM_1_ and DM_2_ are mounted on flip mounts to enable realignment of the system with the laser beam and the white light beam.

1. Prior to aligning the 4π-cavity, ensure that the excitation beam is parallel to the white light and laser beams used to align the cavity

2. Following the alignment of PS-M, place DM_1_ and align the beam to the base of the cavity

3. Following the alignment of the entire 4π-cavity, place DM_2_. Note that the beam existing the cavity through the transparent half of the double-sided mirror sample and DM_2_ should be parallel to the beam existing BS towards TL and C

4. Attach an excitation filter to the laser

*III. Alignment of the camera and imaging optics*

TL and C are positioned and aligned following the alignment of the entire system.

1. Place TL and C on an optical rail

2. Attach a standard mirror on the mount of O_1_ and block the beam exiting O_2_ (as the double-sided mirror is still at the sample holder)

3. Use the laser to illuminate BS with low power and image the point-spread function of TL on C

4. Move TL until the narrowest point-spread function is observed on C

5. Attach an emission filter on C

*Routine alignment procedure*

Prior to each particle tracking experiment, a fringe pattern should be observed with the excitation laser using an immobile fluorescent beads sample (Materials and methods). For consistency, ensure that the fluorescently coated facet of the sample is closer to PS-M.

1. Image the immobile fluorescent beads using O_1_ and O_2_ on C. Two overlapping images should be observed

2. Move PS-M to observe fringes

3. If fringes are properly observed, move PS-M back to its zero phase shift position

4. If fringes are not visible, move the sample together with the two objectives forward and backward until fringes are properly observed

5. If fringes are still not seen, replace the sample with the double-sided mirror sample and observe for fringes with the white light beam. Once the white light fringes are properly observed, reinsert the immobile fluorescent beads sample and repeat steps 1-4
